# Effects of Extrusion on Protein Textures of Hydrolysed Rice and Pea Isolates

**DOI:** 10.3390/foods14213590

**Published:** 2025-10-22

**Authors:** Mª Melchora Muñoz, Mª Dolores Garrido, Irene Peñaranda

**Affiliations:** Department of Food Technology, Veterinary Faculty, University of Murcia, Espinardo, 30100 Murcia, Spain; mmm81247@um.es (M.M.M.); mgarrido@um.es (M.D.G.)

**Keywords:** low-moisture extrusion, nozzle diameter, plant-based protein, textured plant protein, meat analogue

## Abstract

The structure and texture of plant proteins play a key role in determining their functionality and use in plant-based meat analogues. Extrusion is an effective technology for developing textures through protein denaturation and alignment, so controlling parameters such as die size is essential, as it determines the pressure, flow and final structure of the product. Conventional meat production faces environmental challenges such as deforestation, greenhouse gas emissions, and high resource use, reinforcing the need for sustainable protein alternatives like plant proteins. The objective of this study was to determine the effect of different nozzle diameters (1 mm and 3 mm) and types of hydrolysed protein isolates (pea and rice) during the extrusion process affect the physicochemical and nutritional parameters of textured proteins. The results indicate that both die diameter and the type of hydrolysed protein isolate significantly influence texturisation. The 1 mm die, by generating higher temperatures and pressures, produces less moist and tougher textures, without achieving the desired fibrous texture. In contrast, the 3 mm die results in moister products with better textural properties, and both showed lower protein losses with the 3 mm nozzle. Moreover, hydrolysed pea protein isolate showed superior textural characteristics and greater water and fat retention. Finally, the combination of the 3 hydrolysed pea protein isolates proves to be the most efficient.

## 1. Introduction

Considering that the world’s population will increase by more than 2000 million people in the next 30 years, it is important to consider a change in the way food is produced, towards other types of systems that are more sustainable, with less environmental impact and greater resilience. The remaining challenge is that it will be necessary to increase the food supply by at least 70% by 2050, which will have a direct impact on the use of arable land, water, and other natural resources, which are increasingly becoming scarce [1].

In this context, the development of plant-based meat analogues has gained considerable attention as part of the global effort to promote sustainable food systems and reduce the environmental footprint of conventional meat production. There is currently a growing trend towards the consumption of plant-based proteins. This has been driven by consumer passion for health and wellness, environmental sustainability, animal welfare, and a flexitarian lifestyle [2]. During the 2020 pandemic, more than $2 billion was invested in plant-based products, which is equivalent to almost half of all the capital invested in this sector in the last forty years. The US retail market for plant-based foods reached a value of $8 billion in 2022, an increase of 7% compared to 2021, despite an adverse economic environment. The global market is projected to increase exponentially to reach $95 billion globally by 2029 [3].

Animal meat is one of the main sources of protein in the human diet. However, the high demand for resources for livestock production does not translate proportionally into the amount of food produced. Livestock farming uses 77% of the world’s agricultural areas, and 41% of global cereal production is used to manufacture animal feed (food: 48%, biofuels: 11%). Therefore, there is limited production of calories and proteins (it produces only 37% of total proteins and 18% of global calories) [4]. It is therefore necessary to implement an effective contraction and convergence policy that addresses environmental, health, social, and ethical issues related to livestock farming [5]. As global meat demand continues to rise due to economic growth, this pressure on production systems reinforces the need to develop sustainable meat substitutes capable of mimicking the sensory and nutritional properties of conventional meat.

The protein source that is mostly used to produce meat analogues is plant-based. The definition of meat analogue refers to the substitution of the main ingredient for something other than meat. It is also called meat substitute, meat alternative, fake or simulated meat, and imitation meat [6].

Several plant sources have been widely studied and used as protein sources, such as legumes (soybeans, peas, beans, and chickpeas) [7,8], cereals (rice, wheat, corn, etc.), and pseudocereals (amaranth, quinoa, and buckwheat) [9]. While recent studies have focused on diverse sources of plant protein, soy protein is still the primary plant protein used to make meat alternatives due to its desired gelling properties and ability to form fibrous matrices [10,11,12,13]. The qualities of soy proteins can be comparable to those of meat, milk, and eggs [14]. The amino acid composition of soy proteins is like that of animal protein, particularly the content of essential amino acids, namely phenylalanine, methionine, threonine, valine, isoleucine, leucine, tryptophan, and lysine [14]. However, soy is a source of allergens, and most of it is genetically modified (GM) [15]. Some challenges posed by soy include that the global supply chain involves long transportation distances, which could lead to environmental problems due to greenhouse gas (GHG) emissions [16], as well as social issues due to food insecurity for local populations [17]. Additionally, ecological problems such as excessive water use [18] and deforestation [19] may arise.

A wide range of plant proteins can serve as potential ingredients for the development of meat analogues and other alternative products [20]. Among these, soy and pea proteins are the most widely used in the production of non-meat products due to their broad availability and relatively low cost [20]. In parallel, rice protein has recently gained importance as an alternative source in the formulation of functional foods and is considered a cost-effective and accessible raw material for various food applications [21]. Therefore, pea and rice proteins represent promising and economically viable raw materials for the large-scale production of plant-based meat analogues.

For these reasons, pea and rice proteins have emerged as promising alternatives to soy in the formulation of plant-based meat analogues. Pea protein (PP) is a valuable plant protein, notable for being hypoallergenic, non-transgenic, balanced in amino acids, and rich in lysine [22,23]. The percentage of amino acids in PP is balanced, with the content of the other seven types of essential human amino acids, except for methionine, being close to the values recommended by the WHO and the FAO [24], in addition to being easy to digest and absorb. Likewise, the lysine content is higher than that of other plant proteins [25]. Although PP has a high nutritional value, its application in formula foods is limited due to its poor solubility in water and limited functional properties [23,26]. To overcome these obstacles, physical, chemical, and enzymatic modifications have been implemented to improve the properties of PP [25]. It has been reported that physical modifications, such as extrusion, a process in which raw materials are exposed to shear forces, high temperatures, and elevated pressures for a short period of time [27], ensure the protection of the product without significantly altering its nutritional value [28]. In addition, extrusion processing gelatinises starch, increases soluble fibre content and eliminates antinutrients [29]. Also, this process reduces microbial contamination and inactivates enzymes [30].

Similarly, rice protein represents another viable source of plant-based protein. In contrast to soy protein, rice protein does not have a bean-like flavour [31]. It is also an important source of energy and protein in the world and is consumed as a staple food in most Asian countries. Rice protein has unique nutritional and hypoallergenic characteristics as compared to other cereal and legume proteins [32] and is ideal as a hypoallergenic protein source to replace milk and soy infant formulas [32,33]. These different plant sources must be textured to obtain a meat-like texture, for which extrusion is the most suitable method. In this study, commercially available hydrolysed isolates of pea and rice proteins were used as the main raw materials. Extrusion is classified into two types: low moisture (dry; <35% water) and high humidity (humid; >50% water) [34]. Thus, low-moisture textured plant protein has a porous structure and a hard texture due to its expansion and low moisture content, respectively, which requires hydration before ingestion. In contrast, textured plant protein with a high moisture content can be consumed immediately without the need for a hydration process, due to its dense structure and chewy texture generated by cooling and a high moisture content, respectively [31].

To achieve the desired textures, flavours and shapes, it is necessary to adjust the extrusion parameters, such as moisture content, feed speed, screw speed, cylinder temperature, and torque [31], and above all, the diameter of the nozzle, as it is the final stage through which the product is formed. The nozzle can influence the final moisture content of the meat analogue, resulting in products with a fibrous and striated texture [35]. The success of meat analogues is based on the ability to adequately replicate the different properties of real meat [36]. However, the process of producing meat analogues that closely resemble real meat, particularly in terms of texture and mouthfeel, remains a challenge [37]. A thorough understanding of the technological and nutritional properties of textured plant proteins is essential to improve their utilisation in food formulation and the development of new processed products [38]. Proteins are essential macronutrients for human nutrition [39], and the nutritional quality of a protein source varies substantially depending on its bioavailability, purity and processing effects [40]. The proteins in cereal or legume flours present modifications depending on the conditions of the extrusion process. This process denatures and aggregates proteins, reducing their solubility [41]. The native structure is lost, the protein subsequently unfolds, and finally cross-links and aligns, forming longer and more fibrous structures. This mechanism is especially notable in the production of textured proteins [42].

Although several studies have investigated the influence of extrusion parameters such as barrel temperature, screw speed, and moisture content on the texturisation of plant proteins, most research has focused on soy-based systems or high-moisture conditions. However, limited information is available regarding how the combined effects of die diameter, screw speed, and barrel temperature influence the techno-functional and nutritional properties of hydrolysed pea and rice protein isolates during low-moisture extrusion. Understanding these relationships is essential for optimising the processing conditions. Therefore, the objective of this study was to evaluate the effect of different nozzle diameters (1 mm and 3 mm) and types of hydrolysed protein isolates (pea and rice) during low-moisture extrusion on the physicochemical and nutritional characteristics of the resulting textured vegetable proteins (TVPs). This research provides valuable insights into how formulation and processing parameters influence texture development, contributing to the optimisation of plant-based meat analogues and offering the industry alternative soy-free texturised proteins with optimal technological and nutritional properties. Therefore, the objective of this study was to evaluate the effect of different nozzle diameters (1 mm and 3 mm) and types of hydrolysed protein isolates (pea and rice) during low-moisture extrusion on the physicochemical and nutritional characteristics of the resulting textured vegetable proteins (TVPs). This research provides valuable insights into how formulation and processing parameters influence texture development, contributing to the optimisation of plant-based meat analogues and offering the industry alternative soy-free texturised proteins with optimal technological and nutritional properties.

## 2. Materials and Methods

### 2.1. Raw Materials

Hydrolysed pea protein isolate (*Pisum sativum* L.) was purchased from Myvegan (Manchester, UK). The powder had a particle size of 150 µm and a protein content of 80 g/100 g product. Hydrolysed rice protein isolate (*Oryza sativa*) was supplied by Roquette Laisa España S.A. (Valencia, Spain), with an extra-fine particle size and a protein content of 85 g/100 g product.

The hydrolysed protein isolates were selected for their modified molecular structure and functional properties compared with native proteins. The hydrolysis process reduces molecular weight and increases the number of exposed hydrophilic groups, thereby improving protein solubility and having the potential to eliminate antinutritional factors [43,44], which can optimise extrusion performance and promote the development of fibrous textures.

### 2.2. Mixtures and Preparation of Texturised Protein

The selected extrusion conditions were based on previous studies indicating that parameters such as barrel temperature, screw speed, and feed moisture play a decisive role in the development of fibrous structures and the functional properties of meat analogues [45]. However, few studies have examined the effect of die diameter under low-moisture extrusion conditions, particularly in systems using hydrolysed pea and rice protein isolates.

The barrel temperatures (65–110–120 °C), screw speed (150 rpm), and feed moisture content (30%) were selected to simulate typical low-moisture texturisation conditions, while allowing the comparison of two die diameters (1 mm and 3 mm) to evaluate their impact on the physicochemical, nutritional, and energy-demand characteristics of the resulting meat analogues.

These parameters were chosen because they fall within the moderate range reported for low-moisture extrusion (10–40% water), where temperatures between 60 °C and 120 °C promote protein unfolding and alignment without inducing thermal degradation [46,47]. This temperature profile ensures sufficient shear and heat transfer to develop fibrous structures while maintaining protein integrity, as illustrated in the schematic representation of the extrusion process by Hülsebusch et al. [47].

The moisture content of the samples was adjusted to 30% (*w*/*v*), based on previous literature indicating that moisture levels between 30% and 40% are optimal for generating fibrous structures in plant proteins through low- and medium-moisture extrusion processes [48]. A 30% level was selected as a balanced midpoint that enables adequate texturisation and functional stability without exceeding the threshold that would require high-moisture processing. Moreover, content is a crucial factor because it regulates both the structural properties (texture and cohesion) and the physicochemical properties (water retention, stability, pH, and water activity) of meat substitutes [49].

The hydrolysed pea protein isolate (HPPI) and hydrolysed rice protein isolate (HRPI) blends were individually adjusted to achieve a final moisture content of 30%, considering the initial moisture content of HPPI (4.99 g water/100 g sample) and HRPI (3.12 g water/100 g sample). The required volume of water was added manually and mixed thoroughly until a homogeneous consistency was achieved. The hydrated blends were stored under refrigeration until extrusion processing, to promote uniform water absorption by the protein matrix.

For each 1000 g batch, the required quantities were:

• HPPI blend (30% moisture): 749.9 g HPPI + 250.1 g added water.
• HRPI blend (30% moisture): 731.2 g HRPI + 268.8 g added water.

All samples used in the experimental process originated from a single batch per type of raw material, i.e., one batch for HPPI and another for HRPI. From each of these batches, hydration to 30% was carried out, followed by extrusion under the conditions described below. All experiments and post-extrusion analyses were performed in triplicate, except for the Texture Profile Analysis (TPA), which was conducted in quintuplicate.

Then, the samples were introduced into a twin-screw EF 21-20 extruder (TSA Industriales.a.s, Luisago, Italy).

The barrel had a length-to-diameter (L/D) ratio of 20:1 and was equipped with three modular heating zones allowing progressive temperature control (65–110–120 °C). The screw configuration combined conveying and kneading elements to ensure uniform mixing, compression, and shear along the barrel. The die consisted of a cylindrical insert with interchangeable nozzles (1 mm and 3 mm diameter) and a length of 10 mm, designed to provide consistent material flow and pressure build-up.

**Figure 1:**
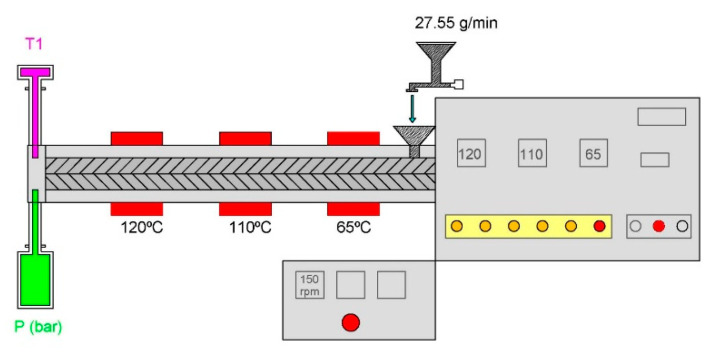
Scheme of conditions used in the extruder.

The conditions used for extrusion (Figure 1) were a 3:1 and 1:1 compression ratio, a dosing speed of 50 rpm (feed rate range, 27.55 g/min), two nozzle diameters (1 mm and 3 mm), 150 rpm of screw rotation, and 65, 110, 120 °C temperature barrel sections. The nozzle outlet temperature for HPPI was 80 °C for the 1 mm nozzle and 76 °C for the 3 mm nozzle, for HRPI it was 95 °C for the 1 mm nozzle and 90 °C for the 3 mm nozzle. The temperatures melted pressure (P), screw speed, feed speed, and torque speed were monitored on the control panel. The extruded products were sealed in polyethylene (PE) bags and stored at −18 °C to prevent microbial growth (e.g., mould development) and to preserve their structural integrity and moisture content prior to analysis. Freezing also minimised oxidative and textural changes, ensuring sample stability and comparability, which were used for subsequent analyses.

### 2.3. Physico-Chemical Properties of Texturised Protein

All analyses were carried out on the texturised samples. A single extruded batch was used for each protein source (HPPI and HRPI). Each analysis was performed in triplicate, except for the texture profile analysis (TPA), which was carried out in quintuplicate.

#### 2.3.1. Instrumental Colour

The colour of the ground texturised samples was measured with a standard D65 illuminant and 10° visual angle (Konica Minolta CM-700d colourimeter, Tokyo, Japan). A reflectance glass (CR-A51, Minolta Camera, Osaka, Japan) was placed between the sample and the colourimeter lens. The measurement window was 6 mm in diameter. The results were expressed using the CIELab system: Chroma, C* (saturation) and hue angle, h*.

#### 2.3.2. Water Content (X_w_)

The water content (X_w_) of the samples was determined by drying the samples until they reached a constant weight at 105 °C in a vacuum oven (BINDER GmbH, Tuttlingen, Germany) according to the AOAC method for extruded samples [50]. From the water content of the samples after extrusion and drying, water losses from the processes of extrusion and drying were calculated.

Moisture content was expressed as g water/100 g sample.

#### 2.3.3. Swelling Index (SWE)

The swelling index (SWE) was measured following the protocol by Robertson et al. [51] based on the bed volume technique. Samples were weighed and transferred to a graduated test tube, and then distilled water was added. The test tubes were maintained for 18 h at ambient temperature (25 °C). The bed volume was measured and expressed as mm of swollen sample per g of the dry initial sample.

#### 2.3.4. Fat Adsorption Index (FAI)

The fat adsorption index (FAI) was determined according to Navarro-González et al. [52], with minor modifications according to Noguerol et al. [53]. A total of 4 g of previously ground textured sample was weighed and mixed with 24 g of sunflower oil in a beaker, incorporating a magnetic stirring bar to facilitate homogeneous mixing. The mixture was shaken for 30 min at 123 rpm using a digital orbital shaker (SHO-1D, Witeg Labortechnik GmbH, Wertheim, Germany), pausing for 30 s every 5 min, removing the beaker from the equipment, to favour a better interaction between the oil and the protein matrix. Then, they were centrifuged at 1600× *g* for 25 min. Free oil was decanted, and FAI was expressed as g oil/g sample.

#### 2.3.5. Texture Profile Analysis (TPA)

The texture profile analysis (TPA) of the protein texturates was carried out using a Texturometer CT310K (Brookfield CNS Engineering Labs. Inc., Harlow, UK) with TexturePro CT V1.8 software, according to the procedures described by Lee and Hong [54] and Wi et al. [55]. A double compression cycle test was performed with a compression of up to 50% of the height of the original portion with a cylindrical probe 10 mm in diameter (TA 10) and a 25 kg load cell. The force–time deformation curves were obtained with a velocity of 2.5 mm/s and a trigger point of 5 g. The parameters that we determined were hardness (g), adhesiveness (mJ), chewiness (mJ), gumminess (g), cohesiveness, elasticity (mm), resilience (J/m^3^), and deformation according to hardness (mm).

#### 2.3.6. Water-Holding Capacity (WHC)

The measurement of water-holding capacity (WHC) was carried out according to the technique by Grau and Hamm [56]. This technique measures the water released by a sample after being subjected to a pressure of 1 kg. For this purpose, 0.3 g of previously ground textured sample was weighed into a watch glass and then transferred to a Whatman No. 540 paper filter (without wrapping the sample). This assembly (sample + Whatman paper filter) was placed between two Petri dishes and subjected to a constant pressure of 1 kg for 10 min. Before pressing, the Whatman paper was kept in an environmental microchamber at 84% relative humidity. The initial weights of the Whatman papers were recorded and, after pressing, the weight of the Whatman paper was measured to determine the percentage of water released from the sample. It was calculated using Formula (1):

(1) $$WHC\;\left(\%\right)=100-\frac{P_{final}-P_{initial}}{P_{sample}}\times 100$$

where

P_final_ = final weight of the paper (g).

P_initial_ = initial weight of the paper (g).

P_sample_ = sample weight (g).

#### 2.3.7. Water Solubility Index (WSI) and Water Absorption Index (WAI)

The water solubility index (WSI) and water absorption index (WAI) were determined with the method by Singh and Smith [57]. The extrudates were first milled to a mean particle size of 180–250 µm. A 2.5 g sample was dispersed in 25 g of distilled water, using a rod to manually break up any lumps. After stirring for 30 min using a magnetic stirrer, the dispersions were rinsed into tared 50 mL centrifuge tubes, made up to 32.5 g, and centrifuged at 3000× *g* for 10 min. The supernatant was decanted for determination of its dissolved solids content, and the sediment was weighed. WAI and WSI were calculated according to Uribe-Wandurraga et al. [58] and Equations (2) and (3).

(2) $$WAI=\frac{Weight\;of\;sediment}{Weight\;of\;dry\;solids}$$

(3) $$WSI\;\left(\%\right)=\frac{Weight\;of\;dissolved\;solids\;in\;supernatant}{Weight\;of\;dry\;solids}\times 100$$

#### 2.3.8. Cooking Loss (CL)

The cooking loss (CL) of protein texturates was calculated based on weight differences according to the method described by Wi et al. [55]. Cooking conditions were set at a temperature of 180 °C on a griddle (Velox CG-1S, Silesia, Barcelona, Spain), until the meat analogue reached an internal temperature of 75 °C. The internal temperature of the meat analogue was measured with a penetration probe. The CL was calculated as the percentage weight difference between the mass before and after cooking, according to Equation (4):

(4) $$CL\;\left(\%\right)=\;\frac{W1-W2}{W1}\;\times \;100$$

where

W1: weight of meat analogue dough (g).

W2: weight of cooked meat analogue (g).

#### 2.3.9. Hygroscopicity (Hy)

Hygroscopicity was determined according to Cai and Corke [59]. The samples remained in contact with an atmosphere with a relative humidity of 91% at 20 °C in a container with saturated Na_2_SO_4_ solution. Samples were weighed after 2 h, and 1, 4, and 7 days, and hygroscopicity was expressed as g of water gained per 100 g of dry solids.

#### 2.3.10. Water Activity

The water activity was analysed according to the determination of water activity ISO 18787:2017 [60].

### 2.4. Nutritional Properties of Texturised Protein

#### 2.4.1. Ash

Ash was evaluated according to method 930.05 of the AOAC procedures [50]. The samples were incinerated at 550 °C at a high pressure in a microwave oven (Muffle Hobersal HK-11, Caldes de Montbui, Spain) for 24 h and the ash was quantified gravimetrically.

#### 2.4.2. Protein

Crude protein content (nitrogen content × 6.25) was assessed using the AOAC method [50].

#### 2.4.3. Fat

Fat determination was performed according to the Soxhlet extraction method ISO 13944:2012 [61].

#### 2.4.4. Carbohydrates

The determination of carbohydrates was carried out according to the FAO/WHO [62] recommendations, based on the results obtained for fat (F), ash (A), protein (P), and moisture (M) content, hence:

(5) Carbohydrates g100 g=100−(F+A+P+M)

### 2.5. Statistical Analysis

For the calculation of mechanical energy and the extrusion parameters, a one-way analysis of variance (ANOVA) was performed, and to evaluate the differences between texturised samples, a two-way ANOVA was applied, using the statistical software SPSS v28 (SPSS, Chicago, IL, USA).

The types of hydrolysed protein isolates (HPPI/HRPI) and nozzle size (1 mm/3 mm) were considered fixed variables, while the three replicates were included as a random factor. The means of the groups were compared using Tukey’s test, considering differences significant at a 95% confidence level (*p* ≤ 0.05). F values were examined to assess the significance of the main effects, and the results are reported as means ± standard deviation.

## 3. Results and Discussion

### 3.1. Protein Texturisation

Table 1 shows the process control parameters for texturing hydrolysed pea/rice protein isolate. Barrel temperature (T_1_) and melt pressure (P) were monitored during extrusion. Specific mechanical energy (SME) can be defined as the energy required to produce 1 g of extrudate [63]. It was calculated [64] from torque (C, N m), screw speed (V, rad s^−1^), and the mass flow rate (Q, g s^−1^) with Equation (6).

(6) $$\mathrm{SME}=\frac{C\times V}{Q}$$

Regarding T_1_ and P, it was observed that T_1_ and P values were significantly greater (*p* ≤ 0.05) for the 1 mm nozzle in the texturisation of rice. This is because it generates a greater compression ratio, leading to higher temperatures and pressures, as the nozzle has a smaller diameter (1 mm).

The SME value of the textured rice sample was significantly higher when using the 1 mm nozzle (*p* ≤ 0.05), followed by the pea texturisation with the same nozzle. As expected, the SME value decreased when using the 3 mm nozzle compared to the 1 mm one, due to the lower pressure and temperature generated by the wider opening, which minimises water evaporation and torque during extrusion. Conversely, in the case of the 1 mm nozzle, the narrower opening results in increased pressure and temperature. This leads to a rise in both torque pressure and nozzle outlet temperature because of the accumulated pressure within the barrel. This situation promotes water evaporation during the extrusion process and, consequently, moisture loss due to the elevated temperatures [65]. As a result, an increase in viscosity and greater resistance of the material to screw movement occurred, implying that more energy is required to push the material through the barrel. Moreover, under both nozzle conditions, the textured pea protein consistently exhibited lower SME values than the textured rice protein, which could be attributed to differences in rheological behaviour and hydration capacity. These factors would result in lower resistance of the material to flow during extrusion, thereby reducing the energy required to form the product structure. This behaviour aligns with the findings reported by Maung et al. [66], who observed that higher moisture combined with a lower screw speed (150 rpm) led to reduced SME, which in turn resulted in lower energy consumption, thereby optimising process efficiency and reducing extrusion-related costs.

**Figure 2:**
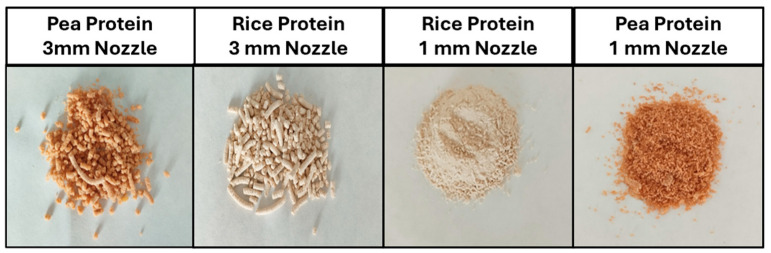
Visual appearance of low-moisture (30%) textured vegetable proteins (TVPs) produced from hydrolysed pea and rice protein isolates with different nozzle diameters (1 mm and 3 mm).

Extrusion integrates various activities, such as transportation and compression, mixing, shearing, plasticising, fusion, cooking, denaturing, fragmentation, texturing, and shaping, among others [67,68]. These operations, which are executed in the extruder, are generated by two Archimedes screws that rotate within the barrel, causing thermal and mechanical stress to the feed material, which promotes physical and chemical changes [67,69]. All these operations affect the loss of the native structure of the proteins, which subsequently unfold and ultimately crosslink and align, forming longer and more fibrous structures, as we can observe in Figure 2.

The products obtained in this study correspond to low-moisture (30%) textured vegetable proteins (TVPs) produced from hydrolysed pea and rice protein isolates. Owing to their fibrous texture and moisture content, these products can be considered final products, like minced plant-based meat, allowing their direct use after gentle cooking or as functional intermediate ingredients in the preparation of other plant-based meat products, such as burgers, sausages, or fillings.

As shown in Figure 2, the nozzle diameter had a significant influence on the macroscopic appearance of the textured proteins. The samples extruded with the 1 mm nozzle (smaller die diameter) experienced higher pressure and shear stress at the die exit, which promoted a greater degree of protein denaturation, alignment, and texturisation, and consequently, a greater expansion upon pressure release. In contrast, the samples obtained with the 3 mm nozzle (larger die diameter) exhibited lower compression and internal pressure, resulting in more limited expansion and a denser, more compact structure.

### 3.2. Functional Characterisation of Textured Protein

Table 2 shows the results of the instrumental colour and mechanical properties. In relation to the type of hydrolysed protein isolate, the results showed significant differences between the two types of hydrolysed protein isolates (*p* ≤ 0.05), with the textured rice having greater luminosity (L*). The higher luminosity (L*) in the textured rice is because the colour of the flour is influenced by the colour of the rice grain, which in this case is white. In the study by Rosniyana et al. [70]. It was observed that the finer the flour, the whiter and brighter the colour.

For parameters a*, b* and C*, significant differences (*p* ≤ 0.05) were observed between the two types of hydrolysed protein isolates, with the textured pea having greater values of a*, b* and C*. This indicates that red/yellow hues were more prevalent in the textured pea, as well as the saturation (C*), which is related to colour intensity. Regarding the parameter hue (h*), significant differences (*p* ≤ 0.05) were observed between the two types of hydrolysed protein isolates, with the textured pea having a higher value of h* for the 1 mm nozzle and the textured rice for the 3 mm nozzle.

As for the effect of the type of nozzle on luminosity (L*), significant differences (*p* ≤ 0.05) were observed in the extrudates made with different nozzles, with the 1 mm nozzles showing the highest L*, regardless of the protein used. These results are in line with those obtained by Oliveira et al. [71], who observed that the conditions of the extrusion process also influenced luminosity; the increase in the temperature led to a higher L* value, and consequently an extruded product with a higher luminosity. Other authors such as Salgado et al. [72], observed that a higher moisture content decreased luminosity, which we could relate to our results, as moisture was lost with the lowest nozzle diameter, which could be related to this increase in brightness. For parameters a*, b*, C*, and h*, significant differences (*p* ≤ 0.05) were observed between the two nozzle diameters used, with higher values found with the texturised pea and rice obtained from the 3 mm nozzle. Therefore, it is observed that with the smaller diameter nozzle (1 mm), colour is lost, thus not achieving the more reddish tones typical of meat proteins, due to the high temperatures and pressures reached in the final section of the extrusion process. Colour is an important attribute of extruded foods, and its changes can provide information about the degree of darkening reactions, such as caramelisation, Maillard reactions, and degree of doneness and pigment degradation that occurs during the extrusion process [73].

For the mechanical properties, the results showed significant differences between the two types of hydrolysed protein isolates (*p* ≤ 0.05), with the textured rice had greater first-bite hardness (HA1). Regarding the parameters of resilience (RE), second-bite hardness (HA2), cohesiveness (CO), and gumminess (GU), significant differences were observed between the two types of proteins (*p* ≤ 0.05), with higher values of resilience (RE), second-bite hardness (HA2), cohesiveness (CO), and gumminess (GU) in the textured pea. In relation to chewiness (CH) and elasticity (EL), the results showed significant differences between the two types of hydrolysed protein isolates (*p* ≤ 0.05), with the textured pea had greater chewiness (CH) and elasticity (EL). Lastly, concerning the effect of the type of hydrolysed protein isolate, no significant differences (*p* ≤ 0.05) were observed in the parameters of deformation according to hardness (DAH) and adhesiveness (AD) for either textured rice or textured pea. Concerning the effect of nozzle type, no significant differences (*p* ≤ 0.05) were observed in the parameters of deformation according to hardness (DAH), adhesiveness (AD), and resilience (RE) for either textured rice or textured pea. For the parameters of second-bite hardness (HA2), cohesiveness (CO), and gumminess (GU), the results showed significant differences (*p* ≤ 0.05) between the two nozzle diameters used, with the textured pea showing greater second-bite hardness (HA2), cohesiveness (CO) and gumminess (GU) for the 1 mm nozzle. Regarding the parameter of first-bite hardness (HA1), significant differences (*p* ≤ 0.05) were detected between the two nozzle diameters employed, with the textured rice demonstrating a greater first-bite hardness for both the 1 mm and 3 mm nozzles. With respect to chewiness (CH), significant differences (*p* ≤ 0.05) were observed between the two nozzle diameters, with a greater chewiness (CH) produced by the 1 mm nozzle for both the textured pea and textured rice. Finally, concerning elasticity (EL), significant differences (*p* ≤ 0.05) were noted between the two nozzle diameters used, with the textured rice had a greater elasticity (EL) for the 1 mm nozzle. The opposite trends in first-bite hardness (HA1) observed between hydrolysed pea and rice protein isolates can be explained by their different hydration behaviours and matrix formation during extrusion. The textured pea protein, with a higher water-holding and absorption capacity, retained more moisture during processing but released it more easily during cooking, resulting in a drier and harder texture. This effect was particularly evident with the 1 mm nozzle, where shear, torque, and temperature were the highest. In contrast, the textured rice protein, with a lower water-holding capacity, formed denser and more compact structures when extruded through the 3 mm nozzle, which promoted greater hardness. This interaction between protein type and nozzle diameter reflects how water retention, moisture distribution, and shear intensity jointly determine the mechanical behaviour of low-moisture extrudates. These effects are directly related to the viscoelastic properties of the molten protein mass during extrusion, which are influenced by the applied thermal and mechanical stresses and, in turn, control protein alignment and the final texture of the product [74].

Therefore, it can be observed that with the 1 mm nozzle, the products exhibited greater hardness, cohesiveness, gumminess, chewiness, and elasticity. This is attributed to the fact that with the smaller diameter nozzle (1 mm), greater pressures and temperatures are experienced, leading to an increased degree of cooking, which may affect the structure of the textured products due to such extreme cooking, which may lead to dense or brittle fibrous structures rather than the well-aligned fibrous texture typical of meat. This coincides with the results obtained by Maung et al. [66], who observed that as the moisture content decreased as a result of high temperatures, the extruded products were harder and chewier. Fan et al. [75] observed the relationship that existed between colour values and texture characteristics, observing that the lowest a* value reflected the hardest sample, in agreement with our results, as the lowest a* values corresponded to the 1 mm nozzle. Zhang et al. [76], explained that the enthalpy changes (ΔH) value measures the crystallinity of starch, and as ΔH increases, the material becomes more crystalline and the mass more viscous. Therefore, as the torque pressure and pressure change (ΔP) increase, the SME becomes larger during the extrusion process. A correlation study was carried out between the extrusion response parameters and the textural characteristics of the extrudates, observing that torque, ΔP, and SME showed a significantly positive correlation with elasticity. Other authors such as Chen et al. [77], found a positive correlation between the SME and the hardness and chewiness of the extrudates. In this context, Fang et al. [78], found that an elevated SME resulted in extrudates with higher tensile strength and hardness, while an increased SME seemed to cause a reduction in the degree of texturing.

Low-moisture textured plant proteins, as in our case, have a porous structure and a hard texture due to their expansion and low moisture content, respectively, which requires hydration before ingestion [31].

**Figure 3:**
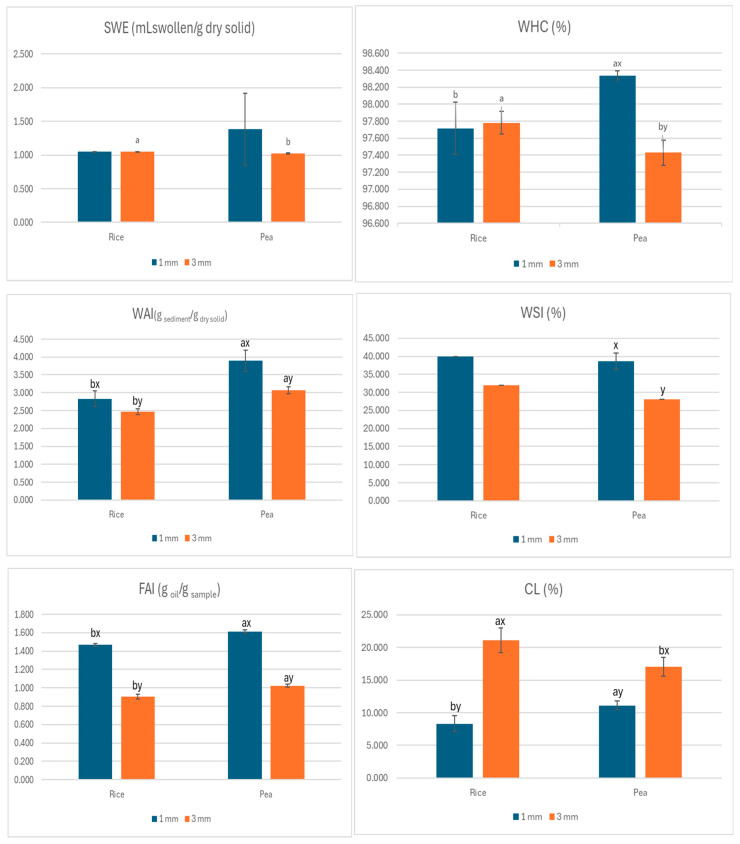
Mean values (and standard deviations) of SWE, WHC, WAI, WSI, FAI and CL. SWE: swelling index (mm of swollen sample/g dry initial sample), WHC: water-holding capacity (%), WAI: water absorption index (g of sediment/g of dry solids), WSI: water solubility index (%), FAI: fat absorption index (g oil/g sample) and CL: cooking loss (%). The means with different superscripts differ significantly at *p* ≤ 0.05, a, b: hydrolysed protein isolate type effect; x, y: nozzle type effect.

Figure 3 shows the behaviour of samples in contact with water (WAI, WSI and SWE), oil (FAI), cooking losses (CL), and water retention capacity (WHC).

The results showed significant differences between the two types of hydrolysed protein isolates (*p* ≤ 0.05), with the textured rice had a higher swelling index (SWE). The high temperature used in extrusion modifies the crystalline structure of starch by breaking intermolecular hydrogen bonds. This increases the sites available for hydrogen bonding, coupled with the release of amylose molecules, making it easier for the starch granules to absorb moisture and expand [79].

Regarding the water holding capacity (WHC), significant differences (*p* ≤ 0.05) were observed between the two types of hydrolysed protein isolates, with rice texturing showing a higher WHC with the 3 mm nozzle, and texturing pea with the 1 mm nozzle. The water holding capacity (WHC) is a fundamental property of meat products. In meat analogues, the WHC measures the ability of proteins to retain water, through the creation of a three-dimensional structure composed of proteins that have joined together, capturing water molecules inside. A higher WHC in meat analogues correlates with improved juiciness [55].

The results indicated significant differences (*p* ≤ 0.05) between the two types of hydrolysed protein isolates, with the textured pea had a higher water absorption index (WAI). The WAI specifically shows the ability of food components, such as starch or fibre, to adhere to water. The temperature of the barrel and the degree of moisture content of the initial ingredient significantly influence the WAI of extruded foods [80].

The results indicated significant differences (*p* ≤ 0.05) between the two types of hydrolysed protein isolates, with the textured pea exhibiting a higher water absorption index (WAI). This parameter reflects the ability of food components, such as starch or fibre, to adhere to water, and is strongly influenced by the temperature of the barrel and the initial moisture content during extrusion [80]. Regarding the water solubility index (WSI), significant differences (*p* ≤ 0.05) were found between the two nozzle diameters applied to the textured pea, with higher WSI values observed for the 1 mm nozzle. This index indicates the extent to which soluble compounds are released from macronutrients [81]. In terms of the fat adsorption index (FAI), the textured pea also showed significantly higher values (*p* ≤ 0.05) compared to textured rice. In addition, significant differences (*p* ≤ 0.05) were observed between both hydrolysed protein isolates with respect to cooking losses (CL), with textured peas showing greater losses for the 1 mm nozzle. However, with the 3 mm nozzle, the trend is reversed, with greater losses observed in textured rice. From a technological point of view, these differences can be considered unfavourable, as greater water loss during cooking can compromise key attributes such as juiciness, texture and yield of the final product. The CL value is directly associated with the degree of shrinkage of the product during cooking and is a key indicator of both its ability to retain water (juiciness) and the yield of the final product [82]. In the specific case of textured peas, greater cooking loss was observed for the 1 mm nozzle, indicating greater water release during the heating process. This lower water retention capacity during cooking could contribute to a drier and potentially harder texture, negatively affecting the juiciness of the final product. This behaviour is consistent with that reported by Chen et al. [83], who indicated that low humidity conditions during extrusion can generate products with a denser and more compact structure, resulting in greater hardness of the final product. Although in our case the moisture content was not modified, the conditions imposed by the 1 mm nozzle (higher pressure and temperature) could have induced comparable technological effects.

In conclusion, the results indicate higher values of WHC, WAI, and FAI for the textured pea, which is due to its higher fibre content as compared to cereals [84], thereby enhancing its oil retention capacity [85] and water retention [86].

In relation to the effect of the type of nozzle, no significant differences were detected (*p* ≤ 0.05) for SWE in rice and textured pea. For WAI and FAI, significant differences were observed based on nozzle type (*p* ≤ 0.05), with higher values of WAI and FAI produced by the 1 mm nozzle for both textured rice and textured pea. Regarding the WHC, significant differences were found according to the type of nozzle (*p* ≤ 0.05), with a larger WHC found in the textured pea with the 1 mm nozzle. Lastly, regarding cooking losses, significant differences (*p* ≤ 0.05) were noted between the two nozzle diameters used, with greater cooking losses (CL) recorded for the 3 mm nozzle for both textured pea and textured rice.

Table 3 shows the mean values and deviations of the moisture content, hygroscopicity and water activity of the samples. Before the protein texturisation process, water is added to hydrate the samples, a significant portion of which is lost during the extrusion process due to the raw material undergoing a combination of various unit operations (compression, mixing, shearing, kneading, high temperature, etc.) [87].

The results showed significant differences (*p* ≤ 0.05) between the hydrolysed protein isolates, with pea texturing having a higher moisture content (X_w_) for the 3 mm nozzle. When the samples were stored for several days in an environment with a relative humidity of 91%, it was observed that most of the textured products, both rice and pea, did not absorb water from the environment. However, on days 1, 4, and 7, the hygroscopicity values of the textured samples showed significant differences (*p* ≤ 0.05) between the two types of hydrolysed protein isolates, with the textured rice had a greater hygroscopicity with the 1 mm nozzle, while the textured pea only absorbed water from the environment on day 4 when processed through the 1 mm nozzle. Lastly, regarding water activity (A_w_), significant differences (*p* ≤ 0.05) were also observed between the hydrolysed protein isolates, with the textured rice showing a higher A_w_ value.

Regarding the effect of nozzle type on the moisture parameter, significant differences (*p* ≤ 0.05) were detected between the two nozzle diameters employed, with higher X_w_ observed in the textured rice for the 3 mm nozzle and in the textured pea for the 3 mm nozzle. In terms of hygroscopicity on days 1, 4, and 7, it was noted that the hygroscopicity values for the textured rice samples were significantly higher for the 1 mm nozzle (*p* ≤ 0.05), with a similar pattern observed in the case of the textured pea on day 4, indicating that these textured samples absorbed water from the environment. Finally, regarding water activity (A_w_), no significant differences (*p* ≤ 0.05) were observed in either the textured rice or the textured pea samples.

The smaller diameter nozzle (1 mm) increases the forming time of the products, resulting in higher temperatures and pressures, which cause evaporation of water during the extrusion process [65]. This justifies the observation that products obtained with the 1 mm nozzle have a lower moisture content, consequently absorbing ambient moisture more rapidly.

### 3.3. Nutritional Value of the Textured Proteins

Table 4 shows the mean values and deviations for proteins, fats, ashes and carbohydrates from the samples.

The results showed significant differences between the two types of hydrolysed protein isolates (*p* ≤ 0.05), with the textured pea presenting a higher ash content, according to the results by Banti et al. for various legumes [88]. Additionally, extrusion may enhance mineral absorption by reducing other factors that inhibit absorption, such as phytic acid, condensed tannins, etc. [30]. Regarding the effect of nozzle type on ash content, significant differences were observed by nozzle type (*p* ≤ 0.05), with the textured pea showing a higher percentage of ash for the 3 mm nozzle.

Finally, regarding the effect of the type of hydrolysed protein isolate and nozzle, in the case of percentage of protein, fat, and carbohydrate content, no significant differences were observed (*p* ≤ 0.05) for either rice texturing or pea texturing.

The fact that the total fat content was not significantly altered during the extrusion may be due to the formation of complexes with amylose or protein [89], which can reduce extractable lipids without changing their total amount. Furthermore, lipids are generally found in reduced quantities in the extrusion of food formulations, as they decrease the friction required for the transmission of mechanical and thermal energy, and in such a situation, they would not play the role of lubricants [80]. Another author [30] observed that lipid levels (˂5%) favour constant extrusion and that the texture is better.

Regarding the protein percentage, we observe that using the smaller diameter nozzle (1 mm) creates a flow restriction, which generates higher pressure and temperature at the outlet of the extruder. This can lead to greater denaturation and degradation of the proteins, which aligns with Verbeek et al. [90], who noted that during extrusion, protein–protein interactions may occur, and these need to be controlled for the processing to be satisfactory, as dense associations and cross-linking occur inside the extruder barrel, resulting in an increase in viscosity and a reduction in chain mobility. Consequently, parameters such as residence time, torque, and pressure in the measurement area increase, leading to protein degradation. Therefore, the results obtained from the protein analysis showed losses in the total protein content of textured rice and pea, related to the diameter of the nozzle used. In the case of the textured pea, an initial protein content of 56 g was used, and after extrusion, contents of 14.69% and 30.36% were observed for the 1 mm and 3 mm nozzles, respectively. This resulted in protein losses of 41.31% with the 1 mm nozzle, and 25.64% with the 3 mm nozzle, thus reflecting a greater loss in the former case.

Similarly, in the analysis of textured rice, with an initial content of 59.5 g of protein, protein percentages after extrusion of 26.06% and 44.59% were obtained for the 1 mm and 3 mm nozzles, respectively. Protein losses were 33.44% for the 1 mm nozzle and 14.91% for the 3 mm nozzle, corroborating the trend observed in the textured pea. Therefore, these results suggest that the use of a larger diameter nozzle (3 mm) may be more favourable to minimise protein losses during the extrusion process, as compared to a smaller diameter nozzle (1 mm).

Finally, regarding the carbohydrate content, although no significant differences were observed, it was found that the values were higher for the smaller diameter nozzle (1 mm). This is due to the higher pressures and temperatures reached because of the restrictions at the nozzle exit hole, which results in a longer shaping time for the texturised products. These conditions could favour phenomena such as starch gelatinisation, by increasing the solubility of the glucan chains [91].

## 4. Conclusions

This study corroborates better results with the 3 mm nozzle and hydrolysed pea protein isolate in the extrusion of plant proteins, given that this combination shows lower specific mechanical energy (SME) values, suggesting a potentially more energy-efficient process while improving texture, moisture, and colour, and increasing the retention capacity of water and fat. On the other hand, the analyses of the nutritional value of the texturised proteins (rice and pea) revealed significant protein losses in both cases, with lower losses when using a 3 mm nozzle. The use of a 1 mm nozzle increases pressure and temperature, promoting the denaturation and degradation of proteins. Regarding ash content, it was higher in the texturised pea with the 3 mm nozzle. In relation to carbohydrates, the 1 mm nozzle resulted in higher values due to a longer shaping time that favours starch gelatinisation. Therefore, the selection of the nozzle diameter and the protein are determining factors when trying to obtain the desired physico-chemical and nutritional properties.

## Figures and Tables

**Table 1:** Mean values (and standard deviations) of barrel temperature (T_1_), melt pressure (P) and specific mechanical energy (SME) of the studied samples.

Sample	Nozzle	T_1_ (°C)	P (Pa)	SME (J/g)
Pea	3 mm	74.33 ± 2.082 ^d^	20.00 ± 4.000 ^d^	1814.80 ± 10.000 ^d^
Rice	3 mm	88.67 ± 1.528 ^b^	24.00 ± 5.000 ^c^	2177.90 ± 19.000 ^c^
Pea	1 mm	78.33 ± 2.082 ^c^	40.00 ± 6.000 ^b^	3629.80 ± 24.000 ^b^
Rice	1 mm	93.33 ± 2.082 ^a^	43.00 ± 5.000 ^a^	3901.90 ± 20.000 ^a^

The same letter in superscript within the column indicates homogeneous groups established by ANOVA (*p* ≤ 0.05).

**Table 2:** Mean values (and standard deviations) of the instrumental colour and mechanical properties of the texturised rice and pea samples.

	Rice		Pea	
Nozzle	1 mm	3 mm	1 mm	3 mm
***Instrumentalcolour***				
L*	98.51 ± 0.264 ^ax^	85.76 ± 0.038 ^ay^	69.18 ± 0.487 ^bx^	66.52 ± 0.444 ^by^
a*	−0.18 ± 0.042 ^by^	1.29 ± 0.012 ^bx^	7.67 ± 0.148 ^ay^	8.19 ± 0.149 ^ax^
b*	1.43 ± 0.239 ^by^	16.19 ± 0.073 ^bx^	19.76 ± 0.287 ^ay^	25.60 ± 0.225 ^ax^
**C**	1.44 ± 0.242 ^by^	16.24 ± 0.073 ^bx^	21.19 ± 0.298 ^ay^	26.88 ± 0.236 ^ax^
**H**	−82.70 ± 0.634 ^by^	85.48 ± 0.053 ^ax^	68.80 ± 0.339 ^ay^	72.29 ± 0.279 ^bx^
***Mechanical properties***				
**HA1**	73.50 ± 7.047 ^by^	235.50 ± 7.188 ^x^	1194.75 ± 329.933 ^ax^	200.25 ± 86.596 ^y^
**DAH**	4.31 ± 1.030	4.65 ± 0.648	4.95 ± 0.095	4.90 ± 0.122
**AD**	0.08 ± 0.045	0.06 ± 0.055	0.08 ± 0.084	0.22 ± 0.228
**RE**	0.04 ± 0.018 ^b^	0.11 ± 0.099	0.09 ± 0.027 ^a^	0.15 ± 0.061
**HA2**	59.00 ± 10.424 ^b^	56.75 ± 19.721	655.75 ± 81.586 ^ax^	91.25 ± 30.956 ^y^
**CO**	0.10 ± 0.050 ^b^	0.05 ± 0.064	0.20 ± 0.079 ^ax^	0.10 ± 0.066 ^y^
**GU**	8.00 ± 2.449 ^b^	6.66 ± 3.786	254.25 ± 49.513 ^ax^	10.00 ± 4.082 ^y^
**CH**	0.10 ± 0.000 ^bx^	0.02 ± 0.030 ^by^	4.45 ± 1.678 ^ax^	0.12 ± 0.096 ^ay^
**EL**	1.15 ± 0.117 ^bx^	0.66 ± 0.063 ^by^	1.64 ± 0.387 ^a^	1.45 ± 0.392 ^a^

L*: lightening, a*: red-green; b*: yellow-blue, C: chroma, H: hue. Mechanical properties: HA1: First-bite hardness (g), AD: adhesiveness (mJ), CH: chewiness (mJ), GU: gumminess (g), CO: cohesiveness, EL: elasticity (mm), RE: resilience (J/m^3^), HA2: second-bite hardness (g) and DAH: deformation according to hardness (mm). The means with different superscripts differ significantly at *p* ≤ 0.05, a, b: hydrolysed protein isolate type effect; x, y: nozzle type effect.

**Table 3:** Mean values (and standard deviations) of water content (X_w_), hygroscopicity (Hy) after 2 h, 1, 4 and 7 days and water activity (A_w_).

Sample	Nozzle	X_w_	Hy_2hrs_	Hy_1d_	Hy_4d_	Hy_7d_	A_w_
Rice	1 mm	10.00 ± 0.000 ^y^	−1.69 ± 0.256 ^ax^	1.96 ± 0.970 ^ax^	3.31 ± 0.597 ^ax^	1.23 ± 0.367 ^ax^	0.68 ± 0.109 ^a^
	3 mm	17.22 ± 0.522 ^bx^	−7.28 ± 0.773 ^by^	−8.81 ± 1.918 ^ay^	−9.76 ± 0.953 ^ay^	−10.35 ± 0.789 ^ay^	0.70 ± 0.134
Pea	1 mm	10.00 ± 0.000 ^y^	−3.78 ± 0.925 ^b^	−1.61 ± 0.600 ^bx^	0.91 ± 0.117 ^bx^	−2.17 ± 0.730 ^bx^	0.56 ± 0.053 ^b^
	3 mm	23.92 ± 0.387 ^ax^	−4.89 ± 0.839 ^a^	−12.771 ± 0.535 ^by^	−16.90 ± 0.676 ^by^	−19.78 ± 0.836 ^by^	0.72 ± 0.293

X_w_: water content (g water/100 g sample), H_y_: hygroscopicity (g water/100 g dry solid), A_w_: water activity (Is a dimensionless parameter ranging from 0 (no available water) to 1 (pure water), indicating the availability of free water in the sample). *The* *means with different superscripts differ significantly at p ≤ 0.05, a, b: hydrolysed protein isolate type effect; x, y: nozzle type effect*.

**Table 4:** Mean values (and standard deviations) of protein, ashes, fat, and carbohydrates.

Sample	Nozzle	Protein	Ashes	Fat	Carbohydrates
Rice	1 mm	26.06 ± 3.848	1.16 ± 0.121 ^b^	0.59 ± 0.195	62.18 ± 3.531
	3 mm	44.59 ± 5.068	1.27 ± 0.012 ^b^	0.67 ± 0.135	60.76 ± 0.142
Pea	1 mm	14.69 ± 5.983	3.19 ± 0.066 ^ay^	0.83 ± 0.227	71.28 ± 5.691
	3 mm	30.36 ± 9.461	4.40 ± 0.167 ^ax^	0.72 ± 0.276	40.79 ± 9.524

Protein (%), ashes (%), fat (% wet matter), carbohydrates (g/100 g). All values are expressed on a wet basis (30% initial hydration). The means with different superscripts differ significantly at *p* ≤ 0.05, a, b: hydrolysed protein isolate type effect; x, y: nozzle type effect.

## Data Availability

The raw data supporting the conclusions of this article will be made available by the authors on request.

## References

[1] McKenzie F.C., Williams J. (2015). Sustainable Food Production: Constraints, Challenges and Choices by 2050. Food Sec..

[2] Green A., Blattmann C., Chen C., Mathys A. (2022). The Role of Alternative Proteins and Future Foods in Sustainable and Contextually-Adapted Flexitarian Diets. Trends Food Sci. Technol..

[3] Guilbeault N. (2024). The Good Eater: A Vegan’s Search for the Future of Food.

[4] Shurtleff W., Aoyagi A. (2022). History of Vegetarianism and Veganism Worldwide (1430 BCE to 1969): Extensively Annotated Bibliography and Sourcebook.

[5] McMichael A.J., Powles J.W., Butler C.D., Uauy R. (2007). Food, Livestock Production, Energy, Climate Change, and Health. Lancet.

[6] Ismail I., Hwang Y.-H., Joo S.-T. (2020). Meat Analog as Future Food: A Review. J. Anim. Sci. Technol..

[7] Coda R., Varis J., Verni M., Rizzello C.G., Katina K. (2017). Improvement of the Protein Quality of Wheat Bread through Faba Bean Sourdough Addition. LWT—Food Sci. Technol..

[8] Tuśnio A., Taciak M., Barszcz M., Święch E., Bachanek I., Skomiał J. (2017). Effect of Replacing Soybean Meal by Raw or Extruded Pea Seeds on Growth Performance and Selected Physiological Parameters of the Ileum and Distal Colon of Pigs. PLoS ONE.

[9] López D.N., Galante M., Robson M., Boeris V., Spelzini D. (2018). Amaranth, Quinoa and Chia Protein Isolates: Physicochemical and Structural Properties. Int. J. Biol. Macromol..

[10] Dou W., Zhang X., Zhao Y., Zhang Y., Jiang L., Sui X. (2022). High Moisture Extrusion Cooking on Soy Proteins: Importance Influence of Gums on Promoting the Fibre Formation. Food Res. Int..

[11] Wang H., Zhang L., Czaja T.P., Bakalis S., Zhang W., Lametsch R. (2022). Structural Characteristics of High-Moisture Extrudates with Oil-in-Water Emulsions. Food Res. Int..

[12] Woo Choi H., Ryoo C., Hahn J., Choi Y.J. (2023). Development of a Novel Technology for High-Moisture Textured Soy Protein Using a Vacuum Packaging and Pressurized Heat (Vacuum-Autoclaving) Treatment. Food Chem..

[13] Schreuders F.K.G., Dekkers B.L., Bodnár I., Erni P., Boom R.M., Van Der Goot A.J. (2019). Comparing Structuring Potential of Pea and Soy Protein with Gluten for Meat Analogue Preparation. J. Food Eng..

[14] Kudełka W., Kowalska M., Popis M. (2021). Quality of Soybean Products in Terms of Essential Amino Acids Composition. Molecules.

[15] Dinani S.T., Van der Harst J.P., Boom R., Van der Goot A.J. (2023). Effect of L-cysteine and L-ascorbic acid addition on properties of meat analogues. Food Hydrocoll..

[16] He R., Zhu D., Chen X., Cao Y., Chen Y., Wang X. (2019). How the Trade Barrier Changes Environmental Costs of Agricultural Production: An Implication Derived from China’s Demand for Soybean Caused by the US-China Trade War. J. Clean. Prod..

[17] Altenburg T. (2007). Donor Approaches to Supporting Pro-Poor Value Chains.

[18] Ercin A.E., Aldaya M.M., Hoekstra A.Y. (2012). The Water Footprint of Soy Milk and Soy Burger and Equivalent Animal Products. Ecol. Indic..

[19] Ferreira M.E., Ferreira L.G., Latrubesse E.M., Miziara F. (2016). Considerations about the Land Use and Conversion Trends in the Savanna Environments of Central Brazil under a Geomorphological Perspective. J. Land Use Sci..

[20] Sha L., Xiong Y.L. (2020). Plant Protein-Based Alternatives of Reconstructed Meat: Science, Technology, and Challenges. Trends Food Sci. Technol..

[21] Roy T., Singh A., Sari T.P., Homroy S. (2023). Rice Protein: Emerging Insights of Extraction, Structural Characteristics, Functionality, and Application in the Food Industry. J. Food Compos. Anal..

[22] Dai T., Li T., Li R., Zhou H., Liu C., Chen J., McClements D.J. (2020). Utilization of Plant-Based Protein-Polyphenol Complexes to Form and Stabilize Emulsions: Pea Proteins and Grape Seed Proanthocyanidins. Food Chem..

[23] Ge J., Sun C., Corke H., Gul K., Gan R., Fang Y. (2020). The Health Benefits, Functional Properties, Modifications, and Applications of Pea (*Pisum sativum* L.) Protein: Current Status, Challenges, and Perspectives. Compr. Rev. Food Sci. Food Saf..

[24] WHO (2007). Protein and Amino Acid Requirements in Human Nutrition: Report of a Joint WHO/FAO/UNU Expert Consultation.

[25] Shuai X., Gao L., Geng Q., Li T., He X., Chen J., Liu C., Dai T. (2022). Effects of Moderate Enzymatic Hydrolysis on Structure and Functional Properties of Pea Protein. Foods.

[26] Lan Y., Xu M., Ohm J.-B., Chen B., Rao J. (2019). Solid Dispersion-Based Spray-Drying Improves Solubility and Mitigates Beany Flavour of Pea Protein Isolate. Food Chem..

[27] Gu B.-J., Kowalski R.J., Ganjyal G.M. (2017). Food Extrusion Processing: An Overview.

[28] Arêas J.A.G., Rocha-Olivieri C.M., Marques M.R. (2016). Extrusion Cooking: Chemical and Nutritional Changes. Encyclopedia of Food and Health.

[29] Sukumar A., Athmaselvi K.A. (2019). Optimization of Process Parameters for the Development of Finger Millet Based Multigrain Extruded Snack Food Fortified with Banana Powder Using RSM. J. Food Sci. Technol..

[30] Choton S., Gupta N., Bandral J.D., Anjum N., Choudary A. (2020). Extrusion Technology and Its Application in Food Processing: A Review. Pharma Innov. J..

[31] Lee J.-S., Oh H., Choi I., Yoon C.S., Han J. (2022). Physico-Chemical Characteristics of Rice Protein-Based Novel Textured Vegetable Proteins as Meat Analogues Produced by Low-Moisture Extrusion Cooking Technology. LWT.

[32] Fiocchi A., Travaini M., D’Auria E., Banderali G., Bernardo L., Riva E. (2003). Tolerance to a Rice Hydrolysate Formula in Children Allergic to Cow’s Milk and Soy. Clin. Exp. Allergy.

[33] Reche M., Pascual C., Fiandor A., Polanco I., Rivero-Urgell M., Chifre R., Johnston S., Martín-Esteban M. (2010). The Effect of a Partially Hydrolysed Formula Based on Rice Protein in the Treatment of Infants with Cow’s Milk Protein Allergy: Hydrolysed Rice Protein Formula in the Treatment of Infants with Cow’s Milk Protein Allergy. Pediatr. Allergy Immunol..

[34] Osen R., Toelstede S., Eisner P., Schweiggert-Weisz U. (2015). Effect of High Moisture Extrusion Cooking on Protein–Protein Interactions of Pea (*Pisum sativum* L.) Protein Isolates. Int. J. Food Sci. Technol..

[35] AINIA Meat Analogues and Textured Protein: What Technology Is Behind These Products?. https://www.ainia.com/ainia-news/analogos-carnicos-proteina-texturizada-tecnologia-productos/.

[36] Zhang S., Huang W., Roopesh M.S., Chen L. (2022). Pre-Treatment by Combining Atmospheric Cold Plasma and pH-Shifting to Prepare Pea Protein Concentrate Powders with Improved Gelling Properties. Food Res. Int..

[37] Lee J.-S., Kim S., Jeong Y.J., Choi I., Han J. (2023). Impact of Interactions between Soy and Pea Proteins on Quality Characteristics of High-Moisture Meat Analogues Prepared via Extrusion Cooking Process. Food Hydrocoll..

[38] Conde J.M., Del Mar Yust Escobar M., Pedroche Jiménez J.J., Rodríguez F.M., Rodríguez Patino J.M. (2005). Effect of Enzymatic Treatment of Extracted Sunflower Proteins on Solubility, Amino Acid Composition, and Surface Activity. J. Agric. Food Chem..

[39] Adenekan M.K., Fadimu G.J., Odunmbaku L.A., Oke E.K. (2018). Effect of Isolation Techniques on the Characteristics of Pigeon Pea (*Cajanus cajan*) Protein Isolates. Food Sci. Nutr..

[40] Sá A.G.A., Moreno Y.M.F., Carciofi B.A.M. (2020). Food Processing for the Improvement of Plant Proteins Digestibility. Crit. Rev. Food Sci. Nutr..

[41] Silvestre-De-León R., Espinosa-Ramírez J., Heredia-Olea E., Pérez-Carrillo E., Serna-Saldívar S.O. (2020). Biocatalytic Degradation of Proteins and Starch of Extruded Whole Chickpea Flours. Food Bioprocess Technol..

[42] Pismag R.Y., Polo M.P., Hoyos J.L., Bravo J.E., Roa D.F. (2024). Effect of Extrusion Cooking on the Chemical and Nutritional Properties of Instant Flours: A Review. F1000Research.

[43] Cui Q., Sun Y., Zhou Z., Cheng J., Guo M. (2021). Effects of Enzymatic Hydrolysis on Physicochemical Properties and Solubility and Bitterness of Milk Protein Hydrolysates. Foods.

[44] Zheng Z., Li J., Li J., Sun H., Liu Y. (2019). Physicochemical and Antioxidative Characteristics of Black Bean Protein Hydrolysates Obtained from Different Enzymes. Food Hydrocoll..

[45] Zhang Y., Gu B.-J., Hwang N., Ryu G.-H. (2025). Optimization of High-Moisture Meat Analog Production with the Addition of Isolated Mung Bean Protein Using Response Surface Methodology. Foods.

[46] Schmid E.-M., Farahnaky A., Adhikari B., Torley P.J. (2022). High Moisture Extrusion Cooking of Meat Analogs: A Review of Mechanisms of Protein Texturization. Compr. Rev. Food Sci. Food Saf..

[47] Hülsebusch L., Heyn T.R., Amft J., Schwarz K. (2025). Extrusion of Plant Proteins: A Review of Lipid and Protein Oxidation and Their Impact on Functional Properties. Food Chem..

[48] Webb D., Dogan H., Li Y., Alavi S. (2023). Physico-Chemical Properties and Texturization of Pea, Wheat and Soy Proteins Using Extrusion and Their Application in Plant-Based Meat. Foods.

[49] Zhang Y., Ryu G.H. (2023). Effects of Process Variables on the Physicochemical, Textural, and Structural Properties of an Isolated Pea Protein-Based High-Moisture Meat Analog. Foods.

[50] Horwitz W., AOAC International (2006). Official Methods of Analysis of AOAC International.

[51] Robertson J.A., De Monredon F.D., Dysseler P., Guillon F., Amado R., Thibault J.-F. (2000). Hydration Properties of Dietary Fibre and Resistant Starch: A European Collaborative Study. LWT—Food Sci. Technol..

[52] Navarro-González I., García-Valverde V., García-Alonso J., Periago M.J. (2011). Chemical Profile, Functional and Antioxidant Properties of Tomato Peel Fibre. Food Res. Int..

[53] Noguerol A.T., Marta Igual M., Pagán M.J. (2022). Developing Psyllium Fibre Gel-Based Foods: Physicochemical, Nutritional, Optical and Mechanical Properties. Food Hydrocoll..

[54] Lee E.-J., Hong G.-P. (2020). Effects of Microbial Transglutaminase and Alginate on the Water-Binding, Textural and Oil Absorption Properties of Soy Patties. Food Sci. Biotechnol..

[55] Wi G., Bae J., Kim H., Cho Y., Choi M.-J. (2020). Evaluation of the Physicochemical and Structural Properties and the Sensory Characteristics of Meat Analogues Prepared with Various Non-Animal Based Liquid Additives. Foods.

[56] Grau R., Hamm R. (1953). Eine einfache Methode zur Bestimmung der Wasserbindung im Muskel. Naturwissenschaften.

[57] Singh N., Smith A.C. (1997). A Comparison of Wheat Starch, Whole Wheat Meal and Oat Flour in the Extrusion Cooking Process. J. Food Eng..

[58] Uribe-Wandurraga Z.N., Igual M., García-Segovia P., Martínez-Monzó J. (2020). Influence of Microalgae Addition in Formulation on Colour, Texture, and Extrusion Parameters of Corn Snacks. Food Sci. Technol. Int..

[59] Cai Y.Z., Corke H. (2000). Production and Properties of Spray-dried Amaranthus Betacyanin Pigments. J. Food Sci..

[60] (2017). Determination of Water Activity.

[61] (2012). Lubricated Metal-Powder Mixes—Determination of Lubricant Content—Soxhlet Extraction Method.

[62] FAO/WHO (1998). Carbohydrates in Human Nutrition: Report of a Joint FAO/WHO Expert Consultation.

[63] Onwulata C.I., Smith P.W., Konstance R.P., Holsinger V.H. (2001). Incorporation of Whey Products in Extruded Corn, Potato or Rice Snacks. Food Res. Int..

[64] Logié N., Della Valle G., Rolland-Sabaté A., Descamps N., Soulestin J. (2018). How Does Temperature Govern Mechanisms of Starch Changes during Extrusion?. Carbohydr. Polym..

[65] Bisharat G.I., Oikonomopoulou V.P., Panagiotou N.M., Krokida M.K., Maroulis Z.B. (2013). Effect of Extrusion Conditions on the Structural Properties of Corn Extrudates Enriched with Dehydrated Vegetables. Food Res. Int..

[66] Maung T.-T., Gu B.-Y., Ryu G.-H. (2021). Influence of Extrusion Process Parameters on Specific Mechanical Energy and Physical Properties of High-Moisture Meat Analog. Int. J. Food Eng..

[67] Wild F. (2016). Manufacture of Meat Analogues Through High Moisture Extrusion. Reference Module in Food Science.

[68] Rosentrater K.A., Evers A.D. (2018). Extrusion Processing of Pasta and Other Products. Kent’s Technology of Cereals.

[69] Kristiawan M., Della Valle G., Kansou K., Ndiaye A., Vergnes B. (2019). Validation and Use for Product Optimization of a Phenomenological Model of Starch Foods Expansion by Extrusion. J. Food Eng..

[70] Rosniyana A., Khalid K.H., Syed Abdullah S.N. (2016). Characteristics of Local Rice Flour (MR 220) Produced by Wet and Dry Milling Methods. J. Trop. Agric. Food Sci..

[71] Oliveira L.C., Schmiele M., Steel C.J. (2017). Development of Whole Grain Wheat Flour Extruded Cereal and Process Impacts on Color, Expansion, and Dry and Bowl-Life Texture. LWT.

[72] Salgado N., Giraldo G.I., Orrego C.E. (2017). Influence of the Extrusion Operating Conditions on the Antioxidant, Hardness and Color Properties of Extruded Mango. LWT.

[73] Altan A., McCarthy K.L., Maskan M. (2008). Twin-Screw Extrusion of Barley–Grape Pomace Blends: Extrudate Characteristics and Determination of Optimum Processing Conditions. J. Food Eng..

[74] Sui X., Zhang T., Zhang X., Jiang L. (2024). High-Moisture Extrusion of Plant Proteins: Fundamentals of Texturization and Applications. Annu. Rev. Food Sci. Technol..

[75] Fan F.H., Ma Q., Ge J., Peng Q.Y., Riley W.W., Tang S.Z. (2013). Prediction of Texture Characteristics from Extrusion Food Surface Images Using a Computer Vision System and Artificial Neural Networks. J. Food Eng..

[76] Zhang W., Li S., Zhang B., Drago S.R., Zhang J. (2016). Relationships between the Gelatinization of Starches and the Textural Properties of Extruded Texturised Soybean Protein-Starch Systems. J. Food Eng..

[77] Chen F.L., Wei Y.M., Zhang B., Ojokoh A.O. (2010). System Parameters and Product Properties Response of Soybean Protein Extruded at Wide Moisture Range. J. Food Eng..

[78] Fang Y., Zhang B., Wei Y. (2014). Effects of the Specific Mechanical Energy on the Physicochemical Properties of Texturised Soy Protein during High-Moisture Extrusion Cooking. J. Food Eng..

[79] Wang P., Fu Y., Wang L., Saleh A.S.M., Cao H., Xiao Z. (2017). Effect of Enrichment with Stabilized Rice Bran and Extrusion Process on Gelatinization and Retrogradation Properties of Rice Starch. Starch Stärke.

[80] Leonard W., Zhang P., Ying D., Fang Z. (2020). Application of Extrusion Technology in Plant Food Processing Byproducts: An Overview. Compr. Rev. Food Sci. Food Saf..

[81] Rathod R.P., Annapure U.S. (2017). Physicochemical Properties, Protein and Starch Digestibility of Lentil Based Noodle Prepared by Using Extrusion Processing. LWT.

[82] Sakai K., Sato Y., Okada M., Yamaguchi S. (2021). Improved Functional Properties of Meat Analogs by Laccase Catalyzed Protein and Pectin Crosslinks. Sci. Rep..

[83] Chen F.L., Wei Y.M., Zhang B. (2011). Chemical Cross-Linking and Molecular Aggregation of Soybean Protein during Extrusion Cooking at Low and High Moisture Content. LWT—Food Sci. Technol..

[84] BEDCA. https://www.bedca.net/.

[85] Dey D., Richter J.K., Ek P., Gu B.-J., Ganjyal G.M. (2021). Utilization of Food Processing By-Products in Extrusion Processing: A Review. Front. Sustain. Food Syst..

[86] Han M., Bertram H.C. (2017). Designing Healthier Comminuted Meat Products: Effect of Dietary Fibres on Water Distribution and Texture of a Fat-Reduced Meat Model System. Meat Sci..

[87] Prabha K., Ghosh P., Joseph R.M., Krishnan R., Rana S.S., Pradhan R.C. (2021). Recent Development, Challenges, and Prospects of Extrusion Technology. Future Foods.

[88] Banti M., Bajo W. (2020). Review on Nutritional Importance and Anti-Nutritional Factors of Legumes. Int. J. Food Sci. Nutr..

[89] Camire M.E., Camire A., Krumhar K. (1990). Chemical and Nutritional Changes in Foods during Extrusion. Crit. Rev. Food Sci. Nutr..

[90] Verbeek C.J.R., Van Den Berg L.E. (2010). Extrusion Processing and Properties of Protein--Based Thermoplastics. Macro Mater. Eng..

[91] Lal M.K., Singh B., Sharma S., Singh M.P., Kumar A. (2021). Glycemic Index of Starchy Crops and Factors Affecting Its Digestibility: A Review. Trends Food Sci. Technol..

